# Control of Centrin Stability by Aurora A

**DOI:** 10.1371/journal.pone.0021291

**Published:** 2011-06-23

**Authors:** Kara B. Lukasiewicz, Tammy M. Greenwood, Vivian C. Negron, Amy K. Bruzek, Jeffrey L. Salisbury, Wilma L. Lingle

**Affiliations:** 1 Department of Biochemistry and Molecular Biology, Mayo Clinic, Rochester, Minnesota, United States of America; 2 Department of Laboratory Medicine and Pathology, Mayo Clinic, Rochester, Minnesota, United States of America; Virginia Tech, United States of America

## Abstract

Aurora A is an oncogenic serine/threonine kinase which can cause cell transformation and centrosome amplification when over-expressed. Human breast tumors show excess Aurora A and phospho-centrin in amplified centrosomes. Here, we show that Aurora A mediates the phosphorylation of and localizes with centrin at the centrosome, with both proteins reaching maximum abundance from prophase through metaphase, followed by their precipitous loss in late stages of mitosis. Over-expression of Aurora A results in excess phospho-centrin and centrosome amplification. In contrast, centrosome amplification is not seen in cells over-expressing Aurora A in the presence of a recombinant centrin mutant lacking the serine phosphorylation site at residue 170. Expression of a kinase dead Aurora A results in a decrease in mitotic index and abrogation of centrin phosphorylation. Finally, a recombinant centrin mutation that mimics centrin phosphorylation increases centrin's stability against APC/C-mediated proteasomal degradation. Taken together, these results suggest that the stability of centrin is regulated in part by Aurora A, and that excess phosphorylated centrin may promote centrosome amplification in cancer.

## Introduction

The centrosome aids in the assembly of the bipolar mitotic spindle and in maintenance of cell polarity. Centrosome abnormalities are observed in many cancers and have been shown to drive chromosomal instability (CIN) and aneuploidy. Several key mitotic kinases, including the Plk, NEK, and Aurora families [1], [2], [3], [4], [5], [6], and elevated levels of phosphorylated centrosomal proteins, including centrin [7], have been implicated in centrosome amplification in cancer.

Aurora A is frequently over-expressed in breast and bladder cancer and its ectopic expression causes centrosome amplification and CIN in cell lines and *in vivo* models [8], [9]. Studies using rat and mouse mammary cancer models demonstrate that Aurora A over-expression and genomic instability are early events in tumor progression [10], [11], [12]. Both Aurora A and the tumor suppressor p53 [13], [14] have been implicated in control of genomic stability and centrosome amplification. Interestingly, phosphorylation of p53 by Aurora A leads to its inactivation and degradation [15], [16].

Centrin, a small EF-hand phospho-protein, is located in the centrosome, pericentriolar material, throughout the cytoplasm, and at times, in the nucleus [17], [18], [19]. Despite its ubiquity, centrin is a reliable marker for centrioles because of its highly focal centriolar concentration [20]. Centrin is essential to centriole duplication, as demonstrated by centriole loss and ultimately cell death when centrin is knocked down [21]. Centrin is phosphorylated at G_2_/M [17], yet little is known about the regulation of centrin stability and abundance during the cell cycle. Because both Aurora A and centrin have been implicated in regulating centrosome structure and function, we hypothesized that posttranslational centrin modifications driven by Aurora A regulate its stability and abundance. Given that centrin is required for centriole duplication, we also investigated whether alterations in centrin stability lead to centrosome amplification.

## Results

**Figure 1:**
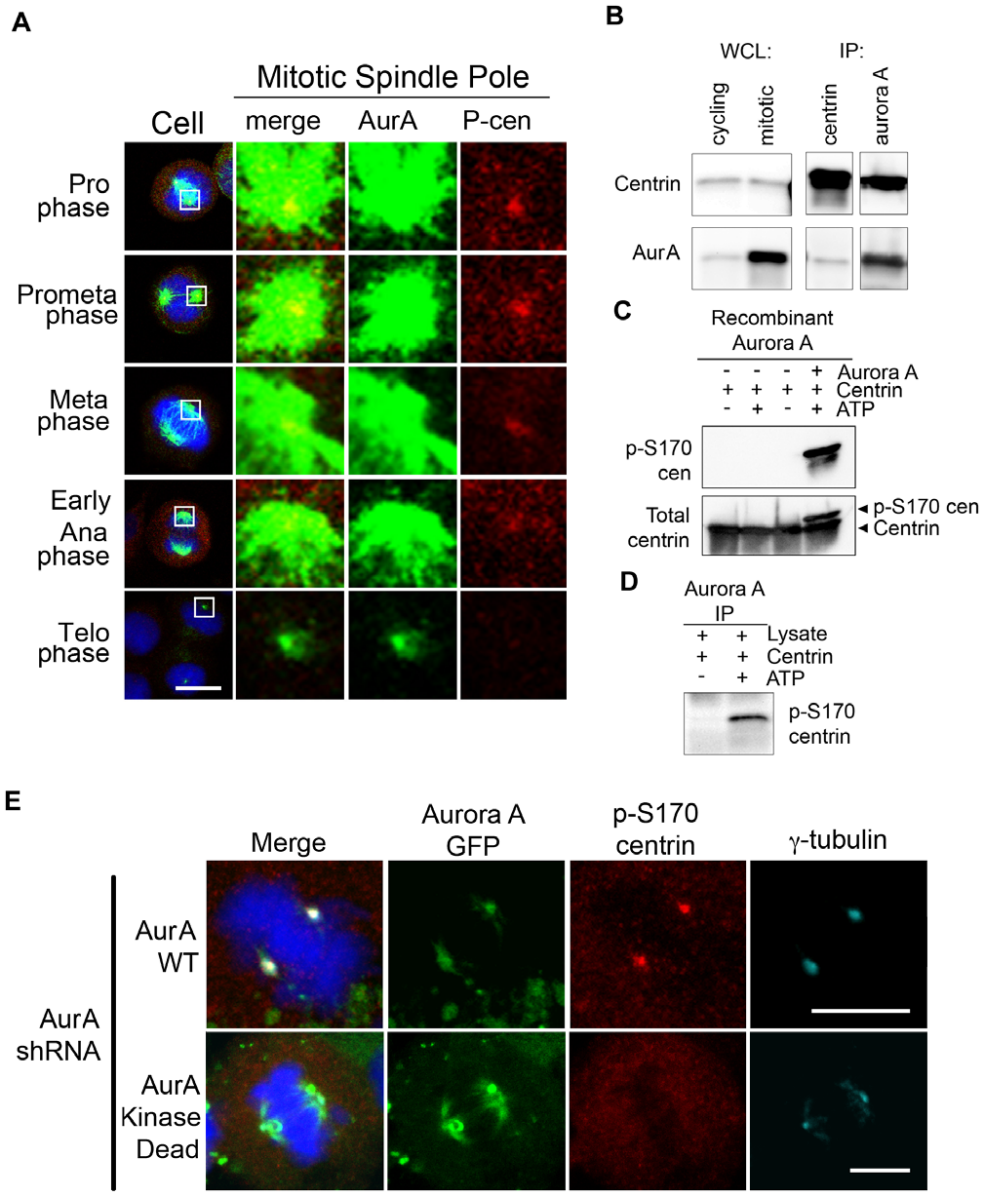
Aurora A localizes with and phosphorylates centrin *in vitro* and in cells. (A) Immunofluorescence confocal microscopy of HeLa cells demonstrates that Aurora A (green) and phospho-S170 centrin (red) both localize at mitotic spindle poles from prophase through metaphase. Phospho-centrin is greatly reduced in anaphase and telophase and Aurora A is greatly reduced by telophase. DNA was counterstained with DAPI (blue). Scale bar = 10 microns. (B) Western blots of whole cell lysates (WCL) from cycling and nocodazole-arrested mitotic cells show that centrin and Aurora A are present in asynchronous and synchronous cultures, with greater amounts of Aurora A in mitotic cells. Reciprocal immunoprecipitations (IPs) of mitotic cell lysates demonstrate an interaction between Aurora A and centrin during mitosis. (C) In *in vitro* kinase assays with recombinant centrin incubated with recombinant Aurora A, centrin was phosphorylated only in the presence of ATP. Under conditions containing ATP, a shift in centrin is detected when Western blotted with a total centrin antibody (lower panel) and phospho-centrin is detected by the phospho-S170 centrin antibody (upper panel). Phospho-centrin is not detected under conditions lacking Aurora A and/or ATP. (D) *In vitro* kinase assays using endogenous Aurora A immunoprecipitated from nocodazole-arrested HeLa cells generated phospho-centrin only in the presence of ATP as demonstrated by Western blotting with the phospho-S170 centrin antibody. (E) Kinase-active Aurora A is required for phosphorylation of centrin at mitotic spindle poles. Cells that lack endogenous Aurora A but express shRNA-resistant GFP-WT Aurora A clearly exhibit phospho-S170 centrin (red) staining; while those that lack endogenous Aurora A and express GFP-kinase dead Aurora A exhibit a nearly complete loss of phospho-S170 centrin (red) staining at the mitotic spindle poles. White arrows denote the focal staining of gamma-tubulin (turquoise) at poles in the cell expressing WT Aurora A or kinase dead Aurora A (green).

We performed immunofluorescence confocal microscopy on HeLa cells stained with antibodies directed against Aurora A and total and phosphorylated-S170 centrin (p-S170 centrin) to determine the localization of p-S170 centrin and Aurora A in intact cells. As demonstrated in Figure 1A, both Aurora A and p-S170 centrin localize at the centrosome from prophase through metaphase. Phospho-centrin, while faintly detectable at some interphase centrosomes (Fig. S4), was most abundant in mitotic cytoplasm and at mitotic spindle poles (Fig. 1A, fourth column). A robust increase in Aurora A at mitotic spindle poles compared to interphase cells (Figures S4+S6) in prophase was accompanied by markedly intense p-S170 centrin staining (Fig. 1A; prophase). This dramatic and specific appearance of p-S170 centrin co-localizing with Aurora A in early prophase cells persisted throughout metaphase. Subsequently p-S170 centrin diminished during anaphase, and by telophase centrosomal and cytosolic p-S170 centrin returned to basal interphase levels (Fig. 1A; metaphase through telophase). Reciprocal immunoprecipitations from double thymidine/nocodazole-synchronized cells demonstrate that Aurora A and centrin both not only localize to the centrosome but can be physically complexed during mitosis (Fig. 1B). Together these experiments show that phosphorylated centrin levels are highest when Aurora A is active [22], [23], and that Aurora A and p-S170 centrin both localize and interact during mitosis.

To determine if this interaction has functional consequences, we performed a kinase assay using recombinant, bacterially-expressed Aurora A as well as Aurora A immunoprecipitated from nocodazole arrested HeLa cell lysates. Both recombinant and immunoprecipitated Aurora A phosphorylate centrin *in vitro*, resulting in a gel shift as detected using a monoclonal antibody against total centrin and using an antibody specific for centrin phosphorylated at S170 (Fig. 1C, D). As a control, we performed an *in vitro* kinase assay solely with centrin and ATP to demonstrate that centrin cannot phosphorylate itself. Mass spectrometry of the excised gel-shifted band from the lane containing: Aurora A, centrin, and ATP confirmed that centrin was phosphorylated at serine 170, as well as at serine 122, in the presence of Aurora A and ATP (data not shown). Both of these sites, (^120^KISF^123^ and ^168^KTSL^171^) match the Aurora A substrate consensus sequence (R/K-x-T/S-I/L/V/F) [24], [25]; in this study we focused on the serine 170 phosphorylation site, which occurs near the carboxy-terminus of centrin.

To further confirm that Aurora A mediates phosphorylation of centrin in cells, we knocked down Aurora A using a plasmid based shRNA directed against the 3′ UTR of the Aurora A mRNA. A Western blot of cell lysates from transfections of the vector control and Aurora A shRNA vector is shown in Figure S2. The mitotic index was reduced to 20% of vector only controls following knock down of Aurora A. In experiments where endogenous Aurora A was knocked down, expression of shRNA-resistant recombinant wildtype Aurora A, but not kinase dead (K162R) Aurora A, rescued the bipolar spindle phenotype and showed intense focal staining with antibodies against gamma-tubulin and p-S170 centrin at the poles of mitotic cells (Fig. 1E). Although bipolar spindles still formed in the presence of kinase dead Aurora A, we noted that the spindle poles lacked focal p-S170 centrin when compared to cells expressing shRNA-resistant-GFP-WT Aurora A. These experiments demonstrate that Aurora A is a physiologically relevant kinase that localizes at the centrosome with centrin and phosphorylates centrin at mitotic spindle poles. In addition, cells over-expressing Aurora A have higher level of centrin and phosphorylated centrin than do cells expressing empty vector (Fig. S5).

**Figure 2:**
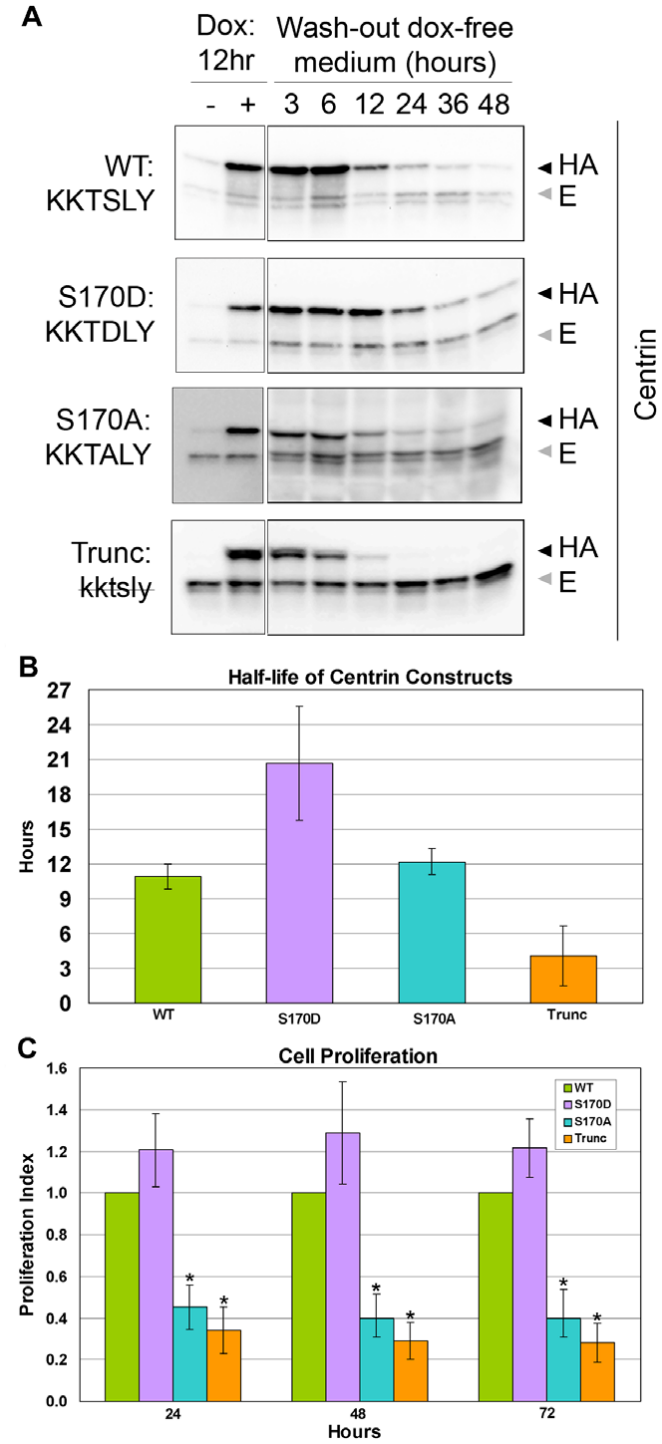
Engineered mutations at S170 affect centrin protein stability and cell proliferation rates in HeLa. (A) Western blotting of whole cell lysates of cells expressing inducible HA-tagged mutant centrin constructs (◀HA) at time points following doxycycline washout reveals that the truncated centrin mutant has the shortest half-life, the S170D mutant that mimics phosphorylated centrin has the longest half-life, while endogenous wildtype centrin (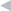
E) remains fairly constant. (B) The half-lives of the centrin mutants were determined by plotting the changes in optical density plots of the Western blots shown in (A) for 5 independent experiments. The S170A and wildtype half-lives are virtually the same at 12.2 and 10.9 hr respectively (p = 0.942). The S170D half-life, at 20.7 hr, is significantly longer than either S170A or wildtype (p<0.027 and 0.013, respectively). In 3 of 5 experiments, the half-life of the truncation mutant could not be determined because the time points measured were not early enough. Results of the 2 experiments in which it could be analyzed indicate the half-life of the truncation mutant is approximately 4.1 hr. (C) Proliferation rates of cells, normalized to HA-tagged wildtype centrin, in which centrin has been knocked down by shRNA and then induced to express only engineered centrin mutants reveal that the truncation mutation greatly reduces the growth rate less than 30% of cells expressing wildtype centrin by 48 hr. Likewise, the S170A mutant (KKT**A**LY) reduces growth rate to approximately 40% at 72 hr. The S170D mutant (KKT**D**LY), which mimics centrin phosphorylated at serine 170, rescues the centrin depletion phenotype to a slightly greater extent than does wildtype centrin. The error bars represent standard deviation of three independent experiments. * indicates p values≤0.03 when compared to wildtype or the S170D centrin construct.

Because the level of phospho-centrin remains high from prophase through metaphase but abruptly decreases at the onset of anaphase (Fig. 1A, anaphase), we sought to determine if the phosphorylation of centrin may play a role in the regulation of centrin stability during the cell cycle. To accomplish this, we established stable HeLa Tet-On cell lines expressing different recombinant HA-tagged centrin mutants that alter the serine residue 170 phosphorylation site: 1) wildtype centrin (^167^KKTSLY^172^, WT); 2) a truncation mutant in which the C-terminal six amino acids of centrin were deleted (^167^kktsly^172^, trunc); 3) a non-phosphorylatable mutant centrin (^167^KKT**A**LY^172^, S170A); and 4) a mutant that mimics constitutive centrin phosphorylation (^167^KKT**D**LY^172^, S170D). Unless otherwise stated, all experiments were performed in the presence of endogenous centrin. In order to assess the half-life of these HA-tagged centrin recombinant products, we performed a “pulse/wash-out” experiment by inducing their expression with doxycycline for 12 hours, followed by washing with and incubation in doxycycline-free medium over a time-course. The half-life of the wildtype HA-centrin (WT), ^167^KKTSLY^172^, and the non-phosphorylatable centrin, ^167^KKT**A**LY^172^ (S170A), were approximately 11 and 12 hours respectively, indicating a near complete turnover once per approximately 24-hour cell cycle period (Fig. 2A, B). The lack of significant differences between the WT and S170A mutant half-lives is likely because only a small pool of centrin (the centrosomal pool) is phosphorylated in a cell cycle dependent fashion. Conversely, the truncation mutant (trunc), ^167^kktsly^172^, was strikingly unstable with a protein half-life of approximately 4.1 hours. Notably, a dramatic increase in half-life of the ^167^KKT**D**LY^172^ (S170D) mutant protein (approximately 21 hours) was observed (Fig. 2A, B). Due to the striking differences in the half-lives of the wildtype and mutant centrins, we performed cell counts to determine if these mutants affect cell proliferation when endogenous centrin is knocked down and the HA-tagged centrins were expressed. As demonstrated by graph in Figure 2C, the S170D centrin expressing cells rescued the centrin depletion phenotype [17] at least as well as, if not better than, the wildtype centrin expressing cells did. On the other hand, the two centrin mutants that were non-phosphorylatable either due to point mutation (S170A) or deletion (kktsly) were not able to rescue the centrin depletion phenotype and proliferated at a much slower rate similar to the rate seen when Aurora A is knocked down (Fig. 2C).

**Figure 3:**
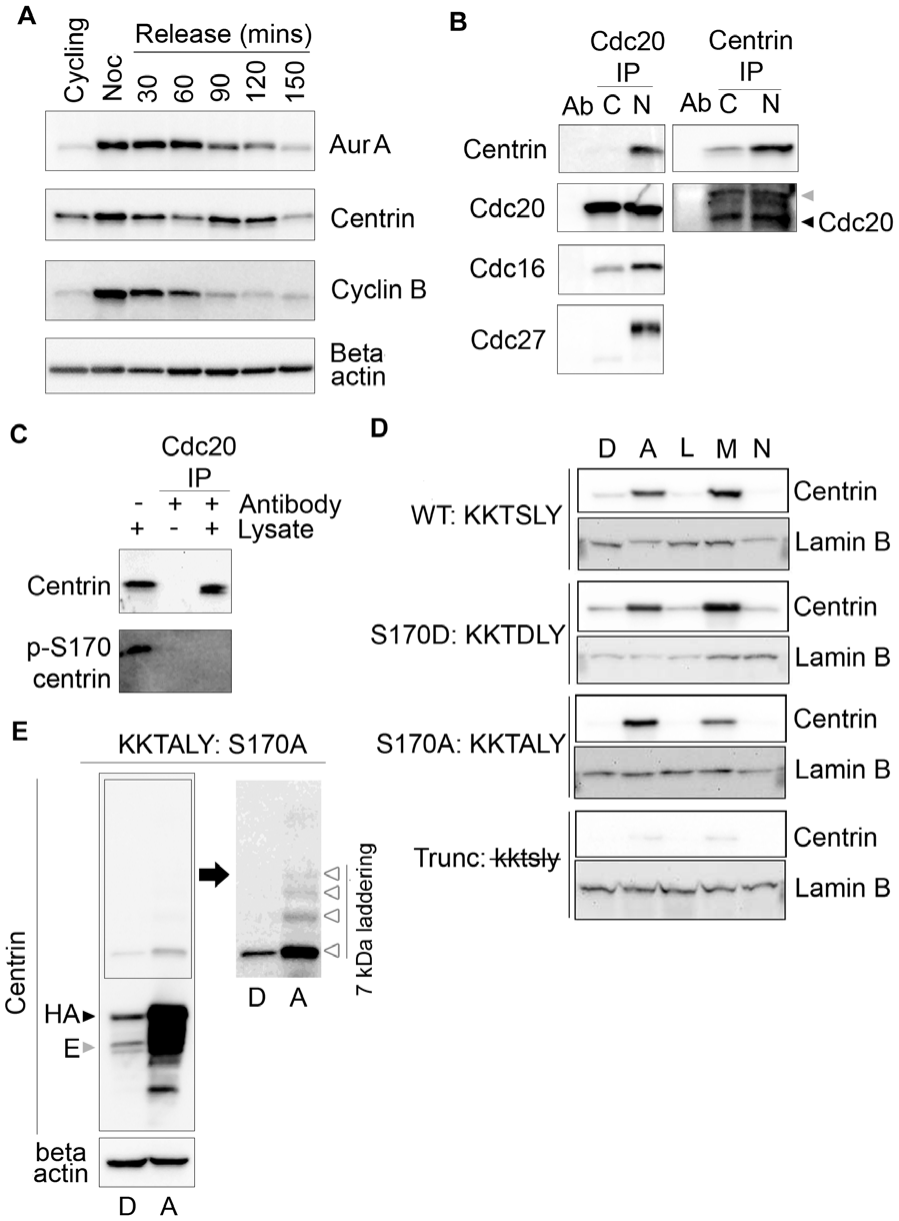
Centrin interacts with APC/C during mitosis. (A) Levels of Aurora A, centrin, and cyclin B were compared in Western blots of lysates of HeLa cells harvested at the indicated time points after synchronization by double thymidine/nocodazole block and release. Cyclin B degradation is used to indicate the onset of anaphase, while the beta-actin blot serves as a loading control. Aurora A and centrin levels drop to basal levels by 150 minutes post-release, while cyclin B is at basal levels by 120 minutes post-release. (B) Equal volumes of immunoprecipitations of lysates from cycling (C) or nocodazole arrested (N) HeLa cells performed with Cdc20 and centrin and Western blotted with the indicated antibodies demonstrate that centrin is pulled down with cdc20 only in nocodazole arrested cells along with Cdc16 and Cdc27. Cdc20 is pulled down with centrin in nocodazole arrested cells and to a slightly lesser extent in cycling cells. The antibody heavy chain and cdc20 were indicated with (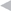) and (◀), respectively. (C) In lysates from asynchronously growing HeLa cells only non-phosphorylated centrin immunoprecipitates with cdc20. Cdc20 does not pull down phosphorylated centrin even though p-S170 centrin is abundant, as seen in the lysate-only lane. (D) HeLa Tet-On cells expressing various centrin mutants treated overnight with DMSO (D), ALLnL (A), leupeptin (L), MG132 (M), and ammonium chloride (N) and Western blotted with antibodies directed against total centrin reveal that DMSO, leupeptin, and ammonium chloride do not prevent centrin degradation, whereas ALLnL and MG132, the two proteasome inhibitors, do prevent degradation of wildtype and mutant forms of centrin. Lamin B loading controls are shown for each mutant cell line. (E) Lysates from HeLa Tet-On cells expressing S170A centrin treated with DMSO (D) or ALLnL (A) for 16 hours were Western blotted for centrin and beta-actin show significant degradation products in the presence of ALLnL but not DMSO. Additionally, when the boxed area of the centrin blot is over-exposed, 7 kDa laddering indicative of ubiquitination is evident (Δ). Endogenous and HA-centrin are indicated with (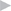) and (▶), respectively.

Due to the abrupt loss of phospho-centrin after anaphase (Fig. 1A), we performed double thymidine/nocodazole-synchronization, shake-off, and release experiments to determine if the total level of centrin fluctuates during cell cycle. Figure 3A illustrates that both Aurora A and centrin abundance peak in G_2_/M (nocodazole) compared to cycling cells and remain high for 60–90 minutes after release from nocodazole treatment. At 120 minutes post-release, Aurora A levels begin to drop; this occurs prior to significant decreases in centrin although there is a slight transient decrease in centrin at 60 minutes post-release. Nonetheless, by 150 minutes both Aurora A and centrin have reached levels comparable to or lower than cycling cells. Cyclin B degradation, which begins 60 minutes after nocodazole release, indicates the physiological onset of metaphase (Fig. 3A). FACS analysis confirms that cell cycles were synchronized and shows cells entering G_1_ as early as 60 minutes after nocodazole release (Fig. S1). This experiment demonstrates temporally coincident expression, accumulation, and post-metaphase decrease of both Aurora A and centrin during the cell cycle.

We reasoned that the decrease in centrin levels could be due to proteasomal degradation because, after peaking in early mitosis, centrin appears to decrease as mitosis progresses to anaphase, when Aurora A and cyclin B also decrease due to proteasome-mediated degradation. The timing of mitotic decrease in centrin protein suggested that centrin could be an anaphase promoting complex/cyclosome (APC/C) substrate. Consistent with this hypothesis, we identified a destruction box (D-box) (^151^RDgdGEVSe^159^) within centrin's protein sequence that closely matches the D-box of the canonical mitotic APC/C substrate cyclin B (http://bioinfo2.weizmann.ac.il/~danag/dbox/form.html). As shown in Figure 3B, centrin immunoprecipitates with cdc20, an activator and substrate binding subunit of the APC/C. Cdc20 immunoprecipitations demonstrate that it interacts with other units of the APC/C, cdc16 and cdc27 (Fig. 3B). Cdc20 immunoprecipitations from asynchronous HeLa cell lysates illustrate that centrin, but not p-S170 centrin, forms a complex with cdc20 (Fig. 3C). These data support the notion that phosphorylated centrin is more stable than unphosphorylated centrin perhaps because the phosphorylated form does not interact with the APC/C protein degradation machinery.

These results led us to further investigate centrin degradation by treating HeLa Tet-On centrin cell lines overnight with a serine protease inhibitor (leupeptin, L), a lysosomal inhibitor (ammonium chloride, N), and two proteasome inhibitors (ALLnL, A and MG132, M), to determine the mechanism of centrin degradation. The lysosome and serine protease inhibitors did not alter the level of centrin in any of the mutants; although the centrin truncation mutant appears to be intrinsically unstable and is likely degraded quickly via an alternative constitutive mechanism (see Discussion). In contrast, the wildtype, S170A, and S170D mutant centrins greatly increased in cells treated with either proteasome inhibitor, indicating that these centrins are degraded via the proteasome (Fig. 3D). Western blots of lysates from HeLa Tet-On cells expressing the S170A centrin mutant treated with the proteasome inhibitor ALLnL demonstrated a laddering of approximately 7 kDa increments when probed with an anti-centrin antibody, consistent with ubiquitination of centrin (Fig. 3E). This laddering, the canonical D-box, and the physical interaction of centrin in a complex containing cdc20, cdc27, and cdc16 suggest that centrin is degraded by ubiquitin-mediated mitosis-specific APC/C activities.

**Figure 4:**
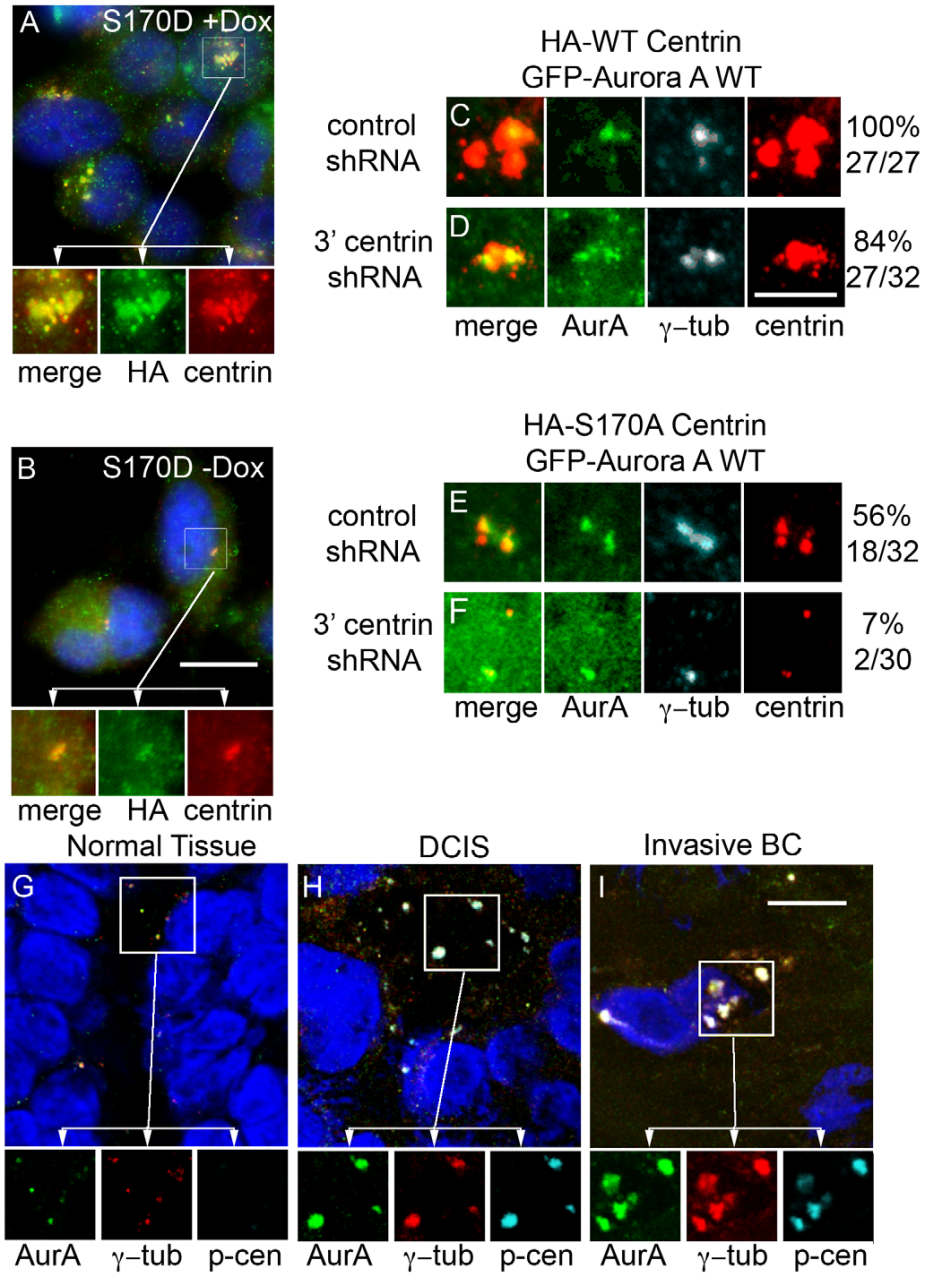
S170D-centrin drives centrosome amplification. (A) Constant expression of S170D-centrin drives centrosome amplification in HeLa Tet-On S170D-centrin cells treated continuously with doxycycline. Cells were stained with an antibody against HA (green) and a polyclonal antibody against centrin (red). Scale bar = 10 microns. (B) Cells that were not induced with doxycycline have normal centrosomes. Cells were stained as in panel (A). Scale bar = 10 microns. (C–F) HeLa Tet-On HA-WT-centrin or HA-S170A-centrin cells transfected with GFP-WT Aurora A and allowed to recover for 24 hours. Cells were then transfected with control shRNA or centrin shRNA and treated with doxycycline to induce HA-centrin expression. Aurora A expression was noted by GFP positivity and cells were stained with centrin (red) and gamma-tubulin (turquoise). A vector control Western blot is shown in Figure S3. Cells over-expressing HA-wildtype centrin in the presence of endogenous centrin have large, numerous centrosomes (C) in virtually all cells. Cells over-expressing HA-wildtype centrin in the absence of endogenous centrin have large, numerous centrosomes (D) in most cells. Conversely cells over-expressing HA-S170A centrin in the presence of endogenous centrin occasionally have slightly enlarged centrosomes (E) and cells over-expressing HA-S170A centrin in the absence of endogenous centrin rarely have slightly enlarged centrosomes (F). GFP positive cells were analyzed for centrosome amplification based on increased size and/or number of centrosomes. The number and percentage of cells with centrosome amplification is presented below each panel. Scale bar = 2.5 microns. (G–I) Frozen breast tissue including normal adjacent breast (G), ductal carcinoma *in situ* (H) and invasive breast cancers (I) were stained with antibodies directed against Aurora A (green), p-S170 centrin (turquoise), and gamma-tubulin (red). Cells were stained with DAPI to mark the DNA. Scale bar = 10 microns.

To investigate the consequences of centrin phosphorylation in cells, endogenous centrin was knocked down using a plasmid based shRNA directed against the 3′ UTR of centrin 2 in HeLa Tet-On cells left untreated (Fig. 4B) or treated with doxycycline to induce expression of the S170D centrin mutant (Fig. 4A). Expression of the S170D mutant resulted in a dramatic increase in HA-tagged centrin staining as compared to untreated cells, and this was accentuated in cells when endogenous centrin was knocked down (Fig. 4A). Detailed electron microscopic studies of the S170D centrin expressing mutant, described elsewhere (Salisbury et al, in preparation), demonstrate that these cells exhibit aberrant centriole-like structures, which may explain the excess centrin staining we observe in Figure 4A. Because this accumulation of centrin resembles centrosome aberrations seen in breast cancer [26] and human breast tumors frequently over-express Aurora A, we extended our studies of phospho-centrin to human breast tumors. Examination of frozen sections of normal breast epithelium adjacent to tumor tissue stained with antibodies directed against Aurora A, gamma-tubulin, and p-S170 centrin show low levels of Aurora A and p-S170 centrin staining at the centrosome (Fig. 4G) consistent with the low proliferative rate of normal breast tissue. In contrast, ductal carcinoma *in situ* (Fig. 4H) and invasive breast tumor tissues (Fig. 4I) displayed both increased size [27] and number of centrosomes (amplified centrosomes or more than two centrosomes per cell) [28], as marked by gamma-tubulin, with striking co-localization of Aurora A and p-S170 centrin. These observations are consistent with what we observed in normal and cancerous breast tissues in our previous study [27]. This is consistent with our observation that expression of the S170D phospho-centrin mutant results in increased centrin-foci in cultured human cells and that Aurora A and phospho-centrin co-localize at amplified centrosomes in human breast tumors.

To determine if the phosphorylation of centrin at serine 170 is necessary for Aurora A-mediated centrosome amplification, we utilized two of the doxycycline HA-tagged centrin mutant expressing HeLa Tet-On cell lines: HA-S170A centrin and HA-WT centrin with the expectation that cells expressing only non-phosphorylatable centrin would not develop amplified centrosomes when compared to cells with phosphorylatable centrin. GFP-WT Aurora A was over-expressed in the HA-WT centrin and HA-S170A centrin cell lines for 24 hours, then the cells were transfected with either control shRNA or 3′ UTR centrin specific shRNA. Four hours after transfection of the shRNA constructs, the cells were induced with doxycycline and allowed to grow for an additional 24 or 48 hours. Cells were then stained for total centrin and gamma-tubulin and analyzed by immunofluorescence confocal microscopy. GFP positive cells were analyzed to ensure we were selecting GFP-WT Aurora A expressing cells. One hundred percent of GFP-WT Aurora A expressing cells exhibited centrosome amplification (Fig. 4C, n = 27). Cells expressing HA-WT centrin in the absence of endogenous centrin also displayed centrosome amplification in the great majority of cells (84% of cells; Fig. 4D, n = 32). Figure S3 shows the expression of HA-centrin while endogenous centrin is knocked down. Approximately half of the cells expressing S170A centrin in the presence of endogenous wildtype centrin exhibited centrosome amplification (56%; Fig. 4E, n = 32), while cells expressing only S170A centrin demonstrated a dramatic decreased in centrosome amplification to 7% (Fig 4F, n = 30). Together with the S170D centrin experiments (Fig. 4A, B), these experiments clearly demonstrate that Aurora A-dependent phosphorylation of centrin is sufficient to cause centrosome amplification and the extent of amplification directly correlates with the expression levels of S170-phosphorylatable centrin.

## Discussion

We previously demonstrated phosphorylation of centrin at serine 170 by PKA [17]. Here we show that Aurora A is another such kinase, which we demonstrate is the relevant mitotic kinase that mediates centrin stability at the time of cell division. This does not preclude a role for PKA in centrin phosphorylation under other physiological circumstances. The precise manner in which excess phosphorylated centrin causes centrosome amplification is not known. One possibility is that the increased stability of centrin directly affects centriole formation through mass action equilibrium binding due to centrin accumulation. Alternatively, p-S170 centrin may adopt a conformation that affects its interaction with other centriole proteins leading to nascent centriole-like structure assembly.

It is also worth noting that centrin's stability is severely affected when the six carboxy-terminal amino acids are deleted (centrin truncation mutant kktsly). These observations coupled with the analysis of the phosphorylation status within this C-terminal tail suggest that these amino acids are essential for proper centrin stability. It is likely that centrin is misfolded without its C-terminal 6 amino acids. Hydrophobic residues are often exposed when proteins are incorrectly folded and this can lead to aggregation of the misfolded proteins. Cells have two main mechanisms for dealing with misfolded proteins: repair and proteolysis [29], [30], [31]. These mechanisms involve both chaperones and the proteasome, but the details of cytosolic protein “quality control”, as compared to endoplasmic reticulum (ER) mechanisms, are currently poorly understood [29], [30], [31]. If refolding of truncated centrin by molecular chaperones, like the heat shock proteins, is not successful, then the truncated centrin would likely be degraded as part of the cytosolic quality control mechanism. Interestingly, the chaperones, Hsp90 and Hsp70, localize to the centrosome and interact with centrin in *Xenopus* [32]. Several published accounts describe the proteasome at the centrosome lending credence to the idea that truncated centrin could be degraded at the centrosome [33], [34]. While it is possible that a truncation mutation of centrin could occur naturally, we chose to focus on the phosphorylation status because we observed centrin phosphorylation in unperturbed cells.

Because both Aurora A and centrin are located at the centrosome and manipulation of either protein can alter the centrosome cycle, we investigated the association between these two proteins. We found that Aurora A and pS170-centrin interact with one another and are both located at the centrosome throughout the cell cycle. Importantly, we show that Aurora A is necessary for the focal localization of phospho-S170-centrin at mitotic spindle poles. Our studies show that Aurora A phosphorylates centrin at serine 170 *in vitro* and that the serine 170 phosphorylation affects the stability of centrin by regulating its interaction with APC/C. Furthermore, we found that over-expression of a centrin mutant that mimics phosphorylation results in accumulation of centriole-like structures in cultured cells and that excess Aurora A and phospho-centrin accumulate at amplified centrosomes of human breast tumors. Expression of the phospho-centrin mimicking mutant can rescue the centrin depletion phenotype, whereas expression of the non-phosphorylatable mutant and the truncation mutant lacking the phosphorylation site cannot rescue the phenotype. Finally we demonstrated that phosphorylation of centrin serine 170 is an absolute requirement for Aurora A-mediated centriole amplification.

Our studies demonstrate that Aurora A mediates phosphorylation of centrin at serine 170 in a cell cycle dependent fashion. Strikingly, the pattern of accumulation and then decrease of phospho-centrin during the course of mitosis is consistent with that observed for many other centrosome-associated proteins. Pronounced accumulation of phosphorylated centrosomal proteins at the onset of prophase has been described previously [35]. Loss of phospho-proteins can be due to the dephosphorylation and/or degradation of phosphorylated proteins. We propose that Aurora A-mediated phosphorylation of centrin at late G_2_/M transiently stabilizes the protein until its dephosphorylation at the metaphase-anaphase transition, allowing for its recognition by the APC/C-machinery, ubiquitination, and consequent proteasomal degradation.

Aurora A can stabilize various proteins via kinase activity-independent and -dependent mechanisms. Both E2F1 and N-myc proteins are stabilized by Aurora A independent of its kinase activity [36], [37]. Aurora A's ability to phosphorylate GSK3beta indirectly protects beta-catenin from GSK3beta-mediated phosphorylation and subsequent proteasomal degradation [38]. Interestingly, Aurora A expression stabilizes ASAP protein levels by preventing its proteasomal degradation, and although, Aurora A directly phosphorylates ASAP, it is unclear if this phosphorylation is responsible for ASAP protein stabilization [39]. Additionally, Aurora A's kinase activity is required for stabilization of cyclin B and its APC/C-mediated proteasomal degradation. But again it is unclear if Aurora A must directly phosphorylate cyclin B for this to occur [40]. Phosphorylation is known to impact the stability of a number of APC/C substrates including cdc6 and Aurora A [41], [42], [43].

Here we show that non-phosphorylated, endogenous centrin interacts with APC/C components cdc20, cdc16, and cdc27, that specific proteasome inhibitors block the degradation of centrin, and that centrin mutated to resemble constitutive phosphorylation at serine 170 (KKT**D**LY, S170D) is more stable than either wildtype or non-phosphorylatable centrin (KKT**A**LY, S170A). Furthermore, kinetic analysis of the degradation of specific centrin mutants shows that wildtype or non-phosphorylatable centrins have a half-life of approximately 11 hours, which is consistent with its degradation once during cell cycle. Interestingly for Aurora A, phosphorylation also plays a protective role against proteasomal degradation [41]. Phosphorylation of Aurora A at serine 51 (S51) protects it from APC/C^cdh1^-mediated ubiquitination and subsequent proteasomal degradation [41]. Similar to centrin, a non-phosphorylatable mutation in Aurora A (S51A) has a shorter protein half-life than wildtype Aurora A [41]. Centrin therefore joins Aurora A and a growing list of cell cycle proteins whose abundance is regulated by phosphorylation and APC/C-mediated destruction.

Proteasomal degradation plays a critical role in regulation of a number of diverse cell cycle components. Among those associated with centrosome behavior are the degradation of cyclin B during metaphase [44], initiation of centrosome separation at G_2_/M [33], and centriole disengagement late in mitosis [45], [46]. Duensing and colleagues demonstrated that treatment of U2OS cells with Z-L_3_VS, which inhibits the trypsin, chymotrypsin, and peptidylglutamyl peptidase activities of the proteasome, led to centrosome over-duplication characterized by a mother centriole surrounded by a rosette of daughter centrioles [1]. These data illustrate that a functional proteasome is essential for centrosome homeostasis. In light of our observations demonstrating that centrin accumulates in the presence of proteasome inhibitors, we speculate that lack of centrin degradation could contribute to the proteasome inhibitor-induced centrosome aberrations.

Interestingly while we were performing revisions on this manuscript, Yang et al. demonstrated that Mps1 phosphorylates centrin-2 at three sites (T45, T47, and T118 or centrin^TTT^) *in vitro* [47]. The authors demonstrate that over-expressing GFP-tagged centrin-2 causes excess centrioles and that non-phosphorylatable centrin mutants, centrin^AAT^ and centrin^TTA^, greatly reduce the over-production of centrioles [47]. Phospho-mimicking centrin mutants, centrin^DDT^, centrin^TTD^, and centrin^DDD^, showed increased formation of excess centrioles, but all three phosphorylation sites were required [47]. Previously the Fisk lab demonstrated that non-degradable centrosomal Mps1 can cause centriole over-production [48]; and in this manuscript, they demonstrate that centrin-2 is required for this process [47]. Interestingly by further studying the over-production of centrioles, Yang et al. demonstrated that over-expression phospho-mimicking centrins mutants can not compensate for a reduction in Mps1 in this process [47]. Because the triple centrin-2 phospho-mimic can't compensate for lack of Mps1, this argues that there are other unidentified phosphorylation sites within centrin-2 or other Mps1 substrates that contribute to this process [47]. These along with other recent findings [49] suggest that centrin-2 regulation including its role in centrosome homeostasis could be far more complicated than originally appreciated.

## Materials and Methods

### Ethics Statement

Frozen breast tissues were collected by the Mayo Clinic Breast SPORE Program under the Mayo Clinic Internal Review Board approval # 08-008233. This IRB-approved Protocol allows for use of tissues collected during standard patient care procedures without patient consent, therefore no consent was obtained. “Continuation of the above referenced study is approved by expedited review procedures (45 CFR 46.110, item 5). The Reviewer determined the research continues to pose no more than minimal risk to subjects. The Reviewer determined that this research continues to satisfy the requirements of 45 CFR 46.111.”

### Reagents

All chemicals were purchased from Sigma (St. Louis, MO) unless otherwise stated.

### Antibodies

Antibodies were purchased from the following sources: monoclonal Aurora A clone 35C1 (AbD Serotec; Raleigh, NC), polyclonal Aurora A, goat polyclonal cdc16, monoclonal and polyclonal cdc20, and monoclonal cdc27 (Santa Cruz Biotechnology Inc.; Santa Cruz, CA), cyclin B (Cell Signaling Technology; Danvers, MA), HA-tag (Covance; Princeton, NJ), and β-actin (Sigma). Monoclonal centrin (20H5), polyclonal centrin (MC1), and p-S170 phospho-centrin antibodies were created in the Salisbury laboratory.

### Cell culture

HeLa cells were maintained in DMEM with 10% FBS, 100 IU/ml penicillin, and 100 µg/ml streptomycin. HeLa Tet-On cells were cultured in DMEM with 10% Tet System Approved FBS (Clontech; Mountain View, CA), 100 IU/ml penicillin, 100 µg/ml streptomycin, 400 µg/ml hygromycin, and 100 µg/ml geneticin. To induce expression of HeLa Tet-On cells containing centrin constructs, doxycycline was added to the medium at a final concentration of 1 µg/ml.

### Cell synchronization techniques

For the double thymidine/nocodazole synchronization, HeLa cells were grown to approximately 80% confluency, treated with 2 mM thymidine for 12–16 hours, and released into medium containing 24 µM deoxycytidine for 9 hours. The second block was done as described above except the deoxycytidine release was only 8 hours. Cells were treated with 0.1 µg/ml nocodazole for six hours, harvested by mitotic shake-off, washed, and allowed to release in nocodazole-free medium. For the nocodazole synchronization, cells were treated with 0.5 µg/ml nocodazole for 12–16 hours and harvested as previously described.

### Western blotting

Cells were lysed in 1% SDS, Tris pH 7.4 lysis buffer supplemented with protease and phosphatase inhibitors and sonicated three times for five seconds/burst. Protein concentration was determined using the BioRad DC Assay. For whole cell lysate Western blotting, 20 µg of protein was separated on 10% or 15% SDS-PAGE gels. After separation, proteins were transferred to PVDF membrane using a semi-dry transfer apparatus (The W.E.P. Company, Seattle, WA), fixed with 0.2% glutaraldehyde for 45 minutes at room temperature, blocked with 5% non-fat dry milk for at least 1 hour at room temperature, and incubated with the indicated antibodies overnight at 4°C. Membranes were incubated in secondary antibodies (GE Healthcare; United Kingdom) diluted to 1∶2500 for one hour at room temperature and visualized with ECL (ECL anti-rabbit or anti-mouse IgG-HRP; GE Healthcare; anti-goat IgG-HRP; Santa Cruz) according to manufacturer's instruction.

### Flow Cytometry

Cells were harvested by trypsinization, fixed in 95% ethanol, incubated on ice for at least one hour, washed three times, and stained with propidium iodide (100 µg/ml in 0.1% sodium citrate). DNA content of cells was determined by measuring the propidium iodide staining on channel FL2 using a Becton Dickinson FACScan (San Jose, CA).

### Immunoprecipitations (IP)

HeLa cells were grown to 85–90% confluence, lysed in 200 µl of mild lysis buffer (1% NP-40, 10 mM EDTA, 10 mM Tris-HCl, pH 7.4 with protease inhibitors and phosphatase inhibitors), scraped and collected into a microfuge tube. Cell lysates were sonicated three times at 5 seconds/bursts. IP buffer A (190 mM NaCl, 50 mM Tris-HCl, 6 mM EDTA, 2.5% Triton X-100, pH 7.4) was added to each tube to bring the cell lysate volume up to 1 ml. Each IP was spun down at 14,000 rpm for 5 minutes, and the supernatant was removed and placed into a new microfuge tube. Six µl of antibody was added to each IP and rocked overnight at 4°C. The next day, 50 µl of Pierce A/G beads (Pierce, Rockford, IL) was added to each IP and allowed to rock at 4°C for 1 hour. IPs were spun down at14,000 rpm for 1 minute to pellet the beads and washed 3 times with IP buffer A and 3 times with IP buffer B (150 mM NaCl, 10 mM Tris-HCl, 5 mM EDTA, 0.1% Triton X-100, pH 7.4). After the final wash, the supernatant was removed and 50 µl of 4× SDS sample buffer were added. Samples were boiled for 5 minutes at 100°C, spun down, and separated by SDS-polyacrylamide gels for Western blotting.

### *In vitro* kinase assay and mass spectrometry

For each reaction, 200 units of recombinant Aurora A (Cell Biosciences; Palo Alto, CA) were incubated with 1 µg recombinant centrin-2 for 30 minutes at 30°C in the presence or absence of 10 mM ATP in kinase buffer (50 mM Tris, 10 mM MgCl2, 0.16 mg/ml DTT, pH 7.4). After the initial 30 minutes, another 10 mM ATP, or equivalent volume of kinase buffer minus ATP, was added and the mixture was allowed to incubate for an additional 30 minutes at 30°C. The reaction was stopped by adding SDS buffer to the samples and boiling the samples. The kinase reactions plus and minus ATP were separated by SDS-PAGE, Coomassie stained, and sent for mass spectrometry analysis to determine phosphorylated residues in centrin.

### Immunofluorescence and microscopy

Cells were grown on coverslips, washed once with MTSB (3 mM EGTA, 50 mM PIPES, 1 mM magnesium sulfate, 25 mM potassium chloride), and fixed in −20°C methanol for 10 minutes. Coverslips were dried at room temperature, rehydrated in 0.05% triton X-100/PBS for 3 minutes, and blocked in goat blocking buffer (5% goat serum, 1% glycerol, 0.1% BSA, 0.1% fish skin gelatin, 0.04% sodium azide, pH 7.2) for at least 30 minutes. Coverslips were incubated in primary antibody for 1 hour at room temperature, washed three times with PBS, incubated in secondary antibody for 1 hour at room temperature, and washed again as above. Coverslips were post-fixed in 4% EM grade paraformaldehyde (Electron Microscopy Sciences, Hatfield, PA) in PBS for five minutes, washed twice, dried, and mounted onto slides with ProLong Gold reagent with DAPI (Invitrogen, Carlsbad, CA). Tissues were treated as described above but were freshly cryosectioned from frozen breast tissue the day of the staining and the MTSB wash step was omitted. Images were collected as Z-stacks on a LSM 510 Confocal Laser Scanning Microscope (Zeiss, Germany) for all images except those in Figure 4A+B, which were collected on a Zeiss Axioskop. To improve their quality, we performed minimal image adjustments on them using PhotoShop as follows. Using “Levels”, both images had their RGB input settings changed to 10, 0.79, and 255. The inset images had blue removed.

The inset images then had the green levels adjusted to minimize the green “haze” from the GFP transfection marker so that the green AlexaFluor 488 signal was clearer. No other post-capture image processing was performed.

### Stable HeLa Tet-On centrin mutant cell lines

The HeLa Tet-On system was purchased from Clontech. Mutations in the centrin-2 sequence, cloned into the pTRE2hygro-HA vector, were created using site-directed mutagenesis. The constructs were transfected into HeLa Tet-On cells containing the pTRE-Tight vector using FuGene 6 (Roche) according to manufacturer's instructions. Stably transfected cells were selected by exposing cells to hygromycin and screened using doxycycline induction.

### Pulse/wash-out experiments and proteasome inhibition

For pulse/wash-out experiments, HeLa Tet-On cells were treated with doxycycline for 12 hours, washed with DMEM medium, then incubated in normal Tet-On growth medium lacking doxycycline. Samples were harvested at indicated times and lysed in SDS lysis buffer as described above. For proteasome inhibition studies, cells were incubated with DMSO as a vehicle control, 100 µM ALLnL or MG132, 20 mM ammonium chloride, or 10 µM leupeptin for at least six hours. Cells were then harvested at indicated times and lysed in SDS lysis buffer containing N-ethylmaleimide (NEM) at a final concentration of 1 mg/ml.

### Expression vector and shRNA construction and knock down of Aurora A and centrin

Aurora A cDNA (NCBI accession # NM_003600.2) was purchased from Origene. It was amplified by standard PCR and cloned into pEGFP-C3 vector (Clontech). shRNA oligos directed against the 3′ UTR of Aurora A (TAGGGATTTGCTTGGGATA) were annealed and cloned into an RNA polymerase III dependent H1 RNA promoter driven vector. The same process was repeated for the shRNA directed against the 3′UTR of centrin-2 (AGCTTTGAGCACCTGCCATTT). HeLa and HeLa Tet-On cells were transfected with the Aurora A expression vectors and/or Aurora A or centrin-2 shRNA using Lipofectamine 2000 (Invitrogen) according to manufacturer's instructions. Doxycycline was added to the cells 4 hours post-transfection and cells were harvested at the indicated times and counted for cell proliferation assays or lysed for Western blot analysis.

### Statistical analysis

Centrin mutant half-life was determined by plotting the optical density of Western blot analyses normalized against beta-actin optical density for each mutant at each time point in 5 independent experiments. A linear trendline was plotted for each curve, and the half-life was determined from the slope of the trendline. The resultant half-life values for each mutant were averaged and the standard deviation and standard error were calculated. The probability that variances between the mutants were not significantly different was determined using an F-test. For the cell proliferation assay probability, a paired Student's T Test was performed on the data normalized against the wildtype results.

## Supporting Information

Figure S1 DNA content of synchronized HeLa cells. HeLa cells harvested at the indicated time points after synchronization by double thymidine/nocodazole block and release. Cells were fixed with ethanol and stained with propidium iodide to measure DNA content.
(DOC)

Figure S2 Aurora A knock down in HeLa cells. HeLa cells were transfected with Aurora A shRNA and whole cell lysates were harvested 24 hours after transfection. Whole cell lysates were separated by SDS-PAGE and blotted with the indicated antibodies.
(DOC)

Figure S3 Centrin knock down in HeLa Tet-On HA-Centrin expressing cells. HeLa cells were transfected with centrin shRNA and whole cell lysates were harvested 48 hours after transfection. Whole cell lysates were separated by SDS-PAGE and blotted with the indicated antibodies. Note that it was possible to induce the expression of HA-centrin (HA) while knocking down endogenous centrin (E).
(DOC)

Figure S4 Asynchronously cycling HeLa cells were fixed, permeabilized, and processed for immunofluorescence as described in the Material and Methods section. This image shows an interphase HeLa cell exhibiting faint p-S170 centrin (red, denoted with white arrowhead) co-localizing with Aurora A (green) staining at the centrosome.
(DOC)

Figure S5 HeLa cells were transfected with either empty pEGFP-C3 or pEGFP-C3-Aurora A constructs and cells were harvested 48 hrs after transfection for immunoprecipitations using anti-centrin and anti-phospho-centrin antibodies. Immunoprecipitations were separated by SDS-PAGE and blotted with anti-centrin antibody. Note that there is more centrin and phospho-centrin in the pEGFP-C3-Aurora A expressing cells.
(DOC)

Figure S6 HeLa cells were processed for immunofluorescence as described in the Material and Methods section. Aurora A was detected with an anti-Aurora A antibody (green) and DNA was counterstained with DAPI (blue). An interphase cell is shown on the left side and an early prophase cell on the right side of the image. Note that Aurora A is much more abundant in the early prophase cell. Scale bar = 10 microns.
(DOC)

## References

[1] Duensing A, Liu Y, Perdreau SA, Kleylein-Sohn J, Nigg EA (2007). Centriole overduplication through the concurrent formation of multiple daughter centrioles at single maternal templates.. Oncogene.

[2] Fry AM, Meraldi P, Nigg EA (1998). A centrosomal function for the human Nek2 protein kinase, a member of the NIMA family of cell cycle regulators.. Embo J.

[3] Liu X, Erikson RL (2002). Activation of Cdc2/cyclin B and inhibition of centrosome amplification in cells depleted of Plk1 by siRNA.. Proc Natl Acad Sci U S A.

[4] Meraldi P, Honda R, Nigg EA (2002). Aurora-A overexpression reveals tetraploidization as a major route to centrosome amplification in p53−/− cells.. Embo J.

[5] Meraldi P, Honda R, Nigg EA (2004). Aurora kinases link chromosome segregation and cell division to cancer susceptibility.. Curr Opin Genet Dev.

[6] Swallow CJ, Ko MA, Siddiqui NU, Hudson JW, Dennis JW (2005). Sak/Plk4 and mitotic fidelity.. Oncogene.

[7] Lingle WL, Lutz WH, Ingle JN, Maihle NJ, Salisbury JL (1998). Centrosome hypertrophy in human breast tumors: implications for genomic stability and cell polarity.. Proc Natl Acad Sci U S A.

[8] Miyoshi Y, Iwao K, Egawa C, Noguchi S (2001). Association of centrosomal kinase STK15/BTAK mRNA expression with chromosomal instability in human breast cancers.. Int J Cancer.

[9] Sen S, Zhou H, Zhang RD, Yoon DS, Vakar-Lopez F (2002). Amplification/overexpression of a mitotic kinase gene in human bladder cancer.. J Natl Cancer Inst.

[10] Goepfert TM, Adigun YE, Zhong L, Gay J, Medina D (2002). Centrosome amplification and overexpression of aurora A are early events in rat mammary carcinogenesis.. Cancer Res.

[11] Li JJ, Weroha SJ, Lingle WL, Papa D, Salisbury JL (2004). Estrogen mediates Aurora-A overexpression, centrosome amplification, chromosomal instability, and breast cancer in female ACI rats.. Proc Natl Acad Sci U S A.

[12] Wang X, Zhou YX, Qiao W, Tominaga Y, Ouchi M (2006). Overexpression of aurora kinase A in mouse mammary epithelium induces genetic instability preceding mammary tumor formation.. Oncogene.

[13] Fukasawa K, Choi T, Kuriyama R, Rulong S, Vande Woude GF (1996). Abnormal centrosome amplification in the absence of p53.. Science.

[14] Winey M (1996). Keeping the centrosome cycle on track. Genome stability.. Curr Biol.

[15] Katayama H, Sasai K, Kawai H, Yuan ZM, Bondaruk J (2004). Phosphorylation by aurora kinase A induces Mdm2-mediated destabilization and inhibition of p53.. Nat Genet.

[16] Liu Q, Kaneko S, Yang L, Feldman RI, Nicosia SV (2004). Aurora-A abrogation of p53 DNA binding and transactivation activity by phosphorylation of serine 215.. J Biol Chem.

[17] Lutz W, Lingle WL, McCormick D, Greenwood TM, Salisbury JL (2001). Phosphorylation of centrin during the cell cycle and its role in centriole separation preceding centrosome duplication.. J Biol Chem.

[18] Paoletti A, Moudjou M, Paintrand M, Salisbury JL, Bornens M (1996). Most of centrin in animal cells is not centrosome-associated and centrosomal centrin is confined to the distal lumen of centrioles.. J Cell Sci.

[19] Acu ID, Liu T, Suino-Powell K, Mooney SM, D'Assoro AB (2010). Coordination of centrosome homeostasis and DNA repair is intact in MCF-7 and disrupted in MDA-MB 231 breast cancer cells.. Cancer Res.

[20] White RA, Pan Z, Salisbury JL (2000). GFP-centrin as a marker for centriole dynamics in living cells.. Microsc Res Tech.

[21] Salisbury JL, Suino KM, Busby R, Springett M (2002). Centrin-2 is required for centriole duplication in mammalian cells.. Curr Biol.

[22] Kimura M, Kotani S, Hattori T, Sumi N, Yoshioka T (1997). Cell cycle-dependent expression and spindle pole localization of a novel human protein kinase, Aik, related to Aurora of Drosophila and yeast Ipl1.. J Biol Chem.

[23] Zhou H, Kuang J, Zhong L, Kuo WL, Gray JW (1998). Tumour amplified kinase STK15/BTAK induces centrosome amplification, aneuploidy and transformation.. Nat Genet.

[24] Cheeseman IM, Anderson S, Jwa M, Green EM, Kang J (2002). Phospho-regulation of kinetochore-microtubule attachments by the Aurora kinase Ipl1p.. Cell.

[25] Ferrari S, Marin O, Pagano MA, Meggio F, Hess D (2005). Aurora-A site specificity: a study with synthetic peptide substrates.. Biochem J.

[26] Lingle WL, Salisbury JL (1999). Altered centrosome structure is associated with abnormal mitoses in human breast tumors.. Am J Pathol.

[27] Lingle WL, Barrett SL, Negron VC, D'Assoro AB, Boeneman K (2002). Centrosome amplification drives chromosomal instability in breast tumor development.. Proc Natl Acad Sci U S A.

[28] Fukasawa K (2007). Oncogenes and tumour suppressors take on centrosomes.. Nat Rev Cancer.

[29] Buchberger A, Bukau B, Sommer T (2010). Protein quality control in the cytosol and the endoplasmic reticulum: brothers in arms.. Mol Cell.

[30] Dobson CM (2003). Protein folding and misfolding.. Nature.

[31] Hartl FU, Hayer-Hartl M (2009). Converging concepts of protein folding in vitro and in vivo.. Nat Struct Mol Biol.

[32] Uzawa M, Grams J, Madden B, Toft D, Salisbury JL (1995). Identification of a complex between centrin and heat shock proteins in CSF-arrested Xenopus oocytes and dissociation of the complex following oocyte activation.. Dev Biol.

[33] Freed E, Lacey KR, Huie P, Lyapina SA, Deshaies RJ (1999). Components of an SCF ubiquitin ligase localize to the centrosome and regulate the centrosome duplication cycle.. Genes Dev.

[34] Wigley WC, Fabunmi RP, Lee MG, Marino CR, Muallem S (1999). Dynamic association of proteasomal machinery with the centrosome.. J Cell Biol.

[35] Vandre DD, Borisy GG (1989). Anaphase onset and dephosphorylation of mitotic phosphoproteins occur concomitantly.. J Cell Sci.

[36] He S, Yang S, Deng G, Liu M, Zhu H (2010). Aurora kinase A induces miR-17–92 cluster through regulation of E2F1 transcription factor.. Cell Mol Life Sci.

[37] Otto T, Horn S, Brockmann M, Eilers U, Schuttrumpf L (2009). Stabilization of N-Myc is a critical function of Aurora A in human neuroblastoma.. Cancer Cell.

[38] Dar AA, Belkhiri A, El-Rifai W (2009). The aurora kinase A regulates GSK-3beta in gastric cancer cells.. Oncogene.

[39] Venoux M, Basbous J, Berthenet C, Prigent C, Fernandez A (2008). ASAP is a novel substrate of the oncogenic mitotic kinase Aurora-A: phosphorylation on Ser625 is essential to spindle formation and mitosis.. Hum Mol Genet.

[40] Qin L, Tong T, Song Y, Xue L, Fan F (2009). Aurora-A interacts with Cyclin B1 and enhances its stability.. Cancer Lett.

[41] Kitajima S, Kudo Y, Ogawa I, Tatsuka M, Kawai H (2007). Constitutive phosphorylation of aurora-a on ser51 induces its stabilization and consequent overexpression in cancer.. PLoS One.

[42] Littlepage LE, Wu H, Andresson T, Deanehan JK, Amundadottir LT (2002). Identification of phosphorylated residues that affect the activity of the mitotic kinase Aurora-A.. Proc Natl Acad Sci U S A.

[43] Mailand N, Diffley JF (2005). CDKs promote DNA replication origin licensing in human cells by protecting Cdc6 from APC/C-dependent proteolysis.. Cell.

[44] Clute P, Pines J (1999). Temporal and spatial control of cyclin B1 destruction in metaphase.. Nat Cell Biol.

[45] Vidwans SJ, Wong ML, O'Farrell PH (1999). Mitotic regulators govern progress through steps in the centrosome duplication cycle.. J Cell Biol.

[46] Tsou MF, Stearns T (2006). Mechanism limiting centrosome duplication to once per cell cycle.. Nature.

[47] Yang CH, Kasbek C, Majumder S, Yusof AM, Fisk HA (2010). Mps1 phosphorylation sites regulate the function of centrin 2 in centriole assembly.. Mol Biol Cell.

[48] Kasbek C, Yang CH, Yusof AM, Chapman HM, Winey M (2007). Preventing the degradation of mps1 at centrosomes is sufficient to cause centrosome reduplication in human cells.. Mol Biol Cell.

[49] Klein UR, Nigg EA (2009). SUMO-dependent regulation of centrin-2.. J Cell Sci.

