# Supplementary material for: Control of Centrin Stability by Aurora A
Source: PLoS One. 2011 Jun 23;6(6):e21291. doi: 10.1371/journal.pone.0021291 (PMC3121746; doi:10.1371/journal.pone.0021291)

**Figure S1**: DNA content of synchronized HeLa cells. HeLa cells harvested at the indicated time points after synchronization by double thymidine/nocodazole block and release. Cells were fixed with ethanol and stained with propidium iodide to measure DNA content.


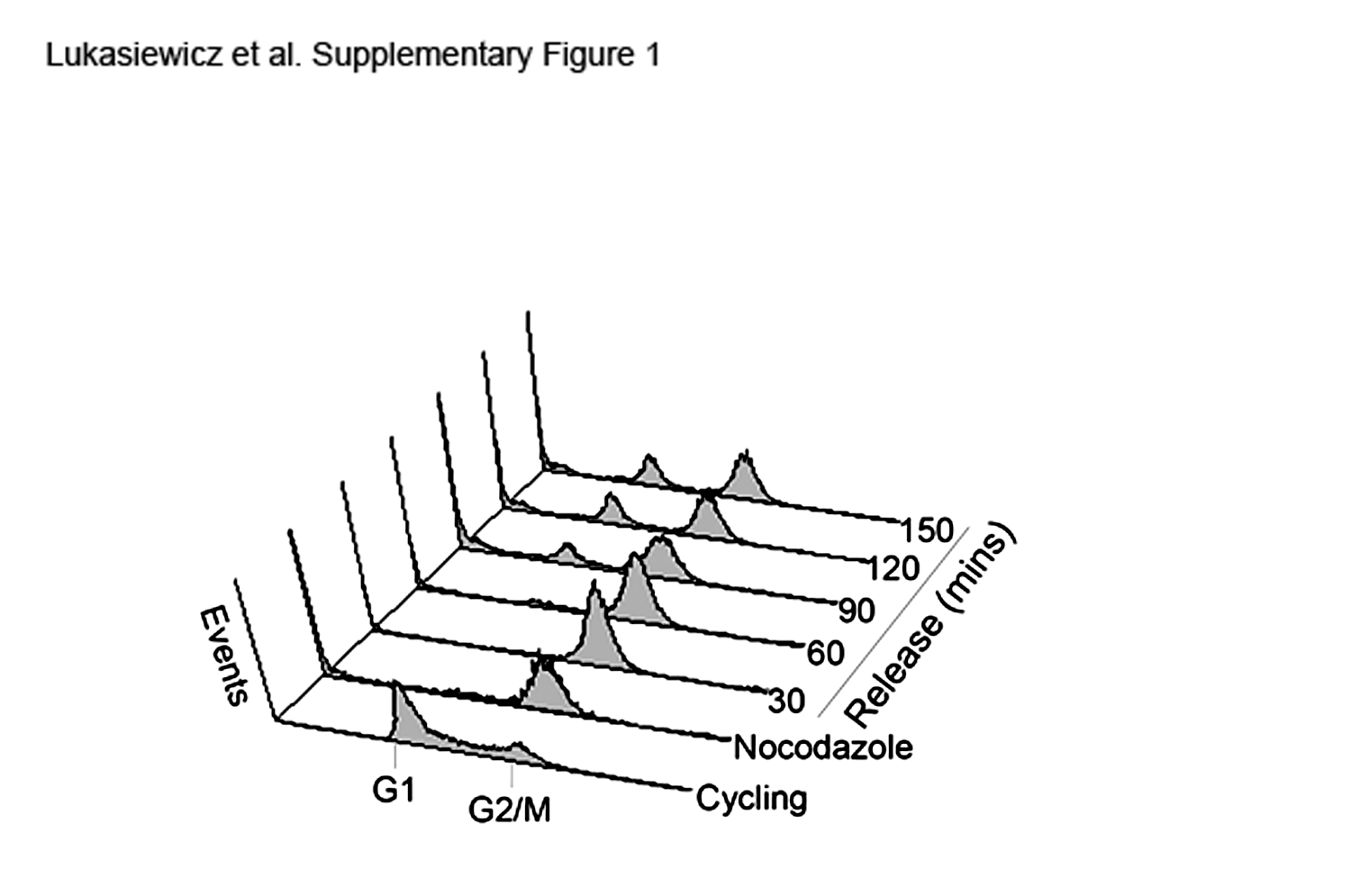

Supplement: Figure S1 — DNA content of synchronized HeLa cells. HeLa cells harvested at the indicated time points after synchronization by double thymidine/nocodazole block and release. Cells were fixed with ethanol and stained with propidium iodide to measure DNA content. (DOC) [file pone.0021291.s001.doc]
