# Supplementary material for: Control of Centrin Stability by Aurora A
Source: PLoS One. 2011 Jun 23;6(6):e21291. doi: 10.1371/journal.pone.0021291 (PMC3121746; doi:10.1371/journal.pone.0021291)

**Figure S2**: Aurora A knock down in HeLa cells. HeLa cells were transfected with Aurora A shRNA and whole cell lysates were harvested 24 hours after transfection. Whole cell lysates were separated by SDS-PAGE and blotted with the indicated antibodies.


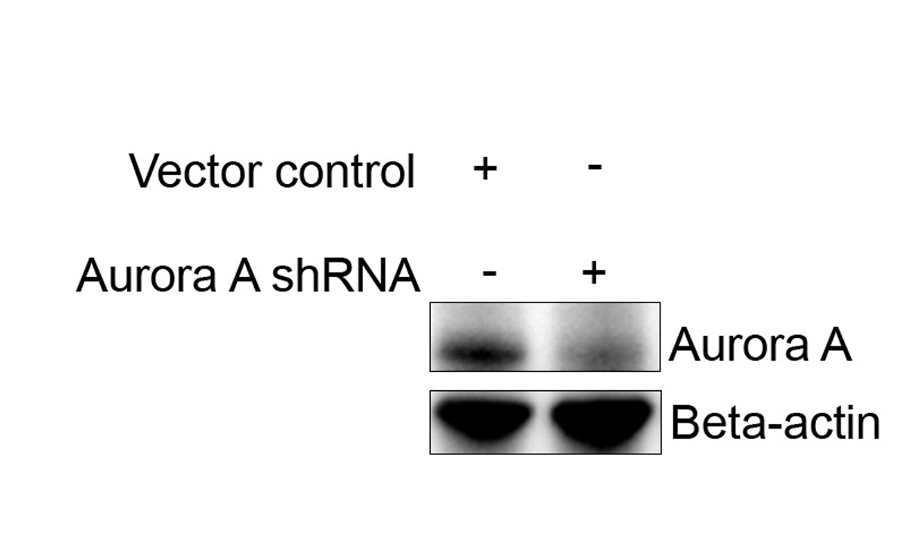

Supplement: Figure S2 — Aurora A knock down in HeLa cells. HeLa cells were transfected with Aurora A shRNA and whole cell lysates were harvested 24 hours after transfection. Whole cell lysates were separated by SDS-PAGE and blotted with the indicated antibodies. (DOC) [file pone.0021291.s002.doc]
