# Supplementary material for: Control of Centrin Stability by Aurora A
Source: PLoS One. 2011 Jun 23;6(6):e21291. doi: 10.1371/journal.pone.0021291 (PMC3121746; doi:10.1371/journal.pone.0021291)

**Figure S3**: Centrin knock down in HeLa Tet-On HA-Centrin expressing cells. HeLa cells were transfected with centrin shRNA and whole cell lysates were harvested 48 hours after transfection. Whole cell lysates were separated by SDS-PAGE and blotted with the indicated antibodies. Note that it was possible to induce the expression of HA-centrin (HA) while knocking down endogenous centrin (E).


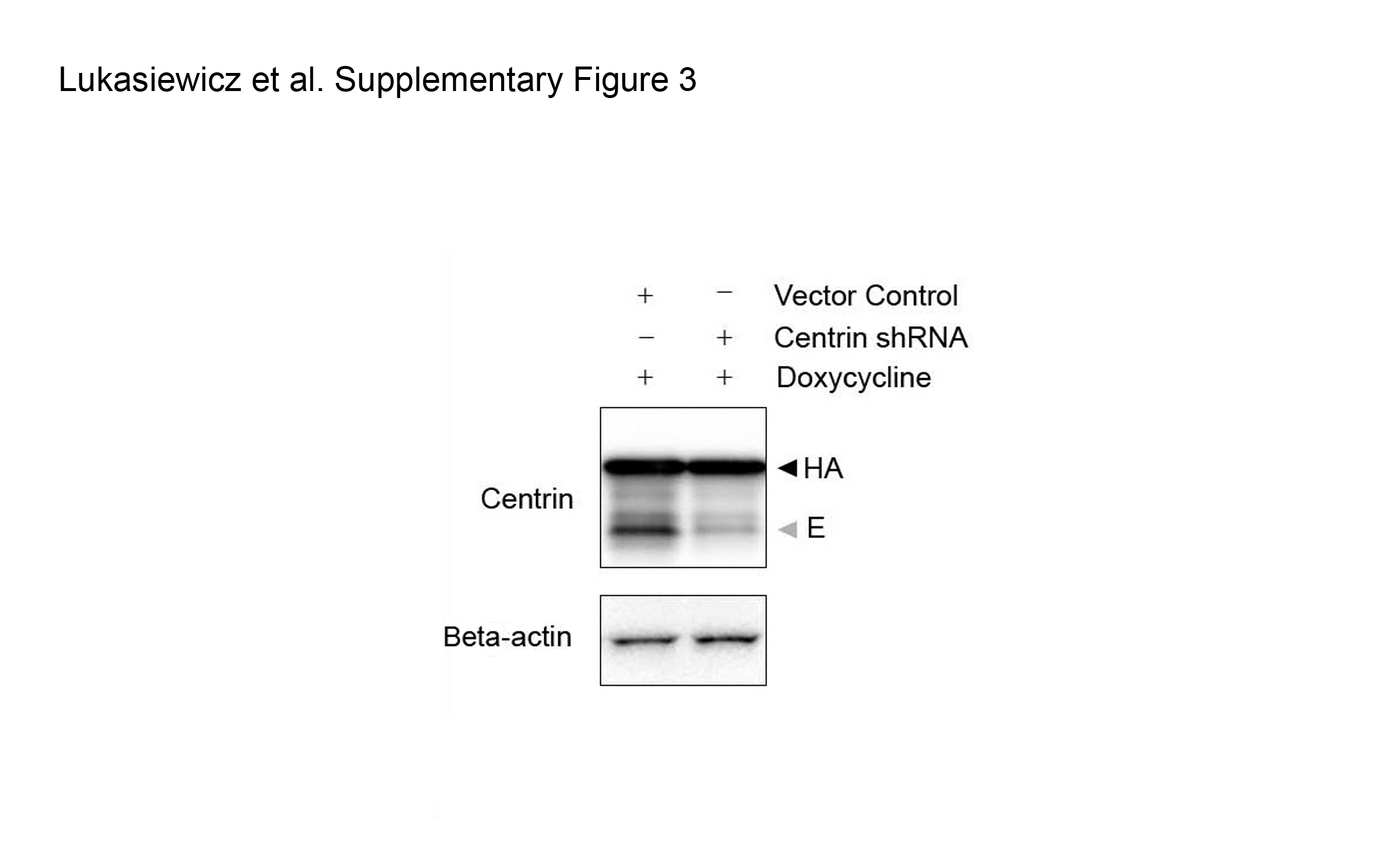

Supplement: Figure S3 — Centrin knock down in HeLa Tet-On HA-Centrin expressing cells. HeLa cells were transfected with centrin shRNA and whole cell lysates were harvested 48 hours after transfection. Whole cell lysates were separated by SDS-PAGE and blotted with the indicated antibodies. Note that it was possible to induce the expression of HA-centrin (HA) while knocking down endogenous centrin (E). (DOC) [file pone.0021291.s003.doc]
