# Supplementary material for: Control of Centrin Stability by Aurora A
Source: PLoS One. 2011 Jun 23;6(6):e21291. doi: 10.1371/journal.pone.0021291 (PMC3121746; doi:10.1371/journal.pone.0021291)

**Figure S4**: Asynchronously cycling HeLa cells were fixed, permeabilized, and processed for immunofluorescence as described in the Material and Methods section. This image shows an interphase HeLa cell exhibiting faint p-S170 centrin (red, denoted with white arrowhead) co-localizing with Aurora A (green) staining at the centrosome.


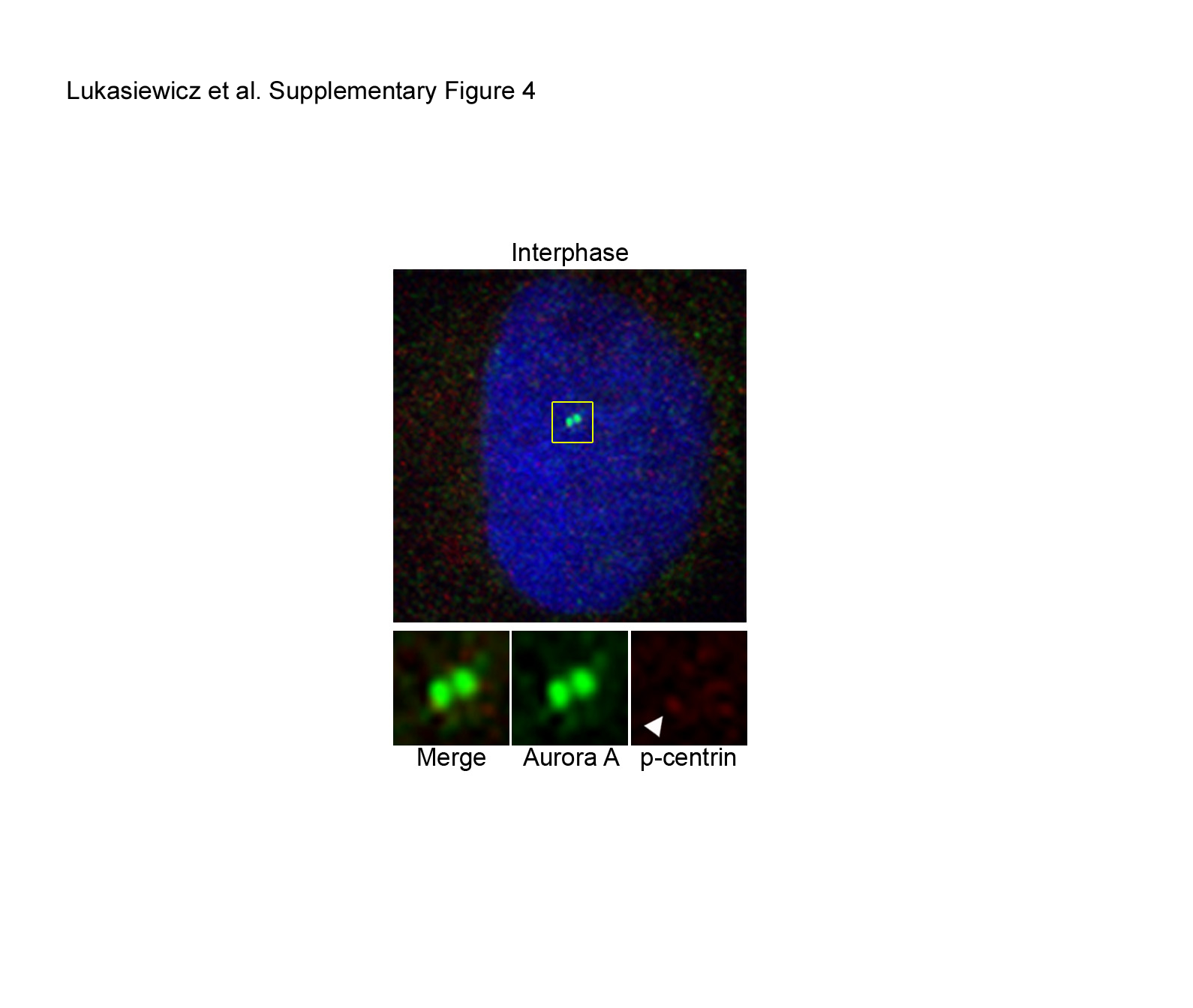

Supplement: Figure S4 — Asynchronously cycling HeLa cells were fixed, permeabilized, and processed for immunofluorescence as described in the Material and Methods section. This image shows an interphase HeLa cell exhibiting faint p-S170 centrin (red, denoted with white arrowhead) co-localizing with Aurora A (green) staining at the centrosome. (DOC) [file pone.0021291.s004.doc]
