# Supplementary material for: Control of Centrin Stability by Aurora A
Source: PLoS One. 2011 Jun 23;6(6):e21291. doi: 10.1371/journal.pone.0021291 (PMC3121746; doi:10.1371/journal.pone.0021291)

**Figure S5**: HeLa cells were transfected with either empty pEGFP-C3 or pEGFP-C3-Aurora A constructs and cells were harvested 48 hrs after transfection for immunoprecipitations using anti-centrin and anti-phospho-centrin antibodies. Immunoprecipitations were separated by SDS-PAGE and blotted with anti-centrin antibody. Note that there is more centrin and phospho-centrin in the pEGFP-C3-Aurora A expressing cells.


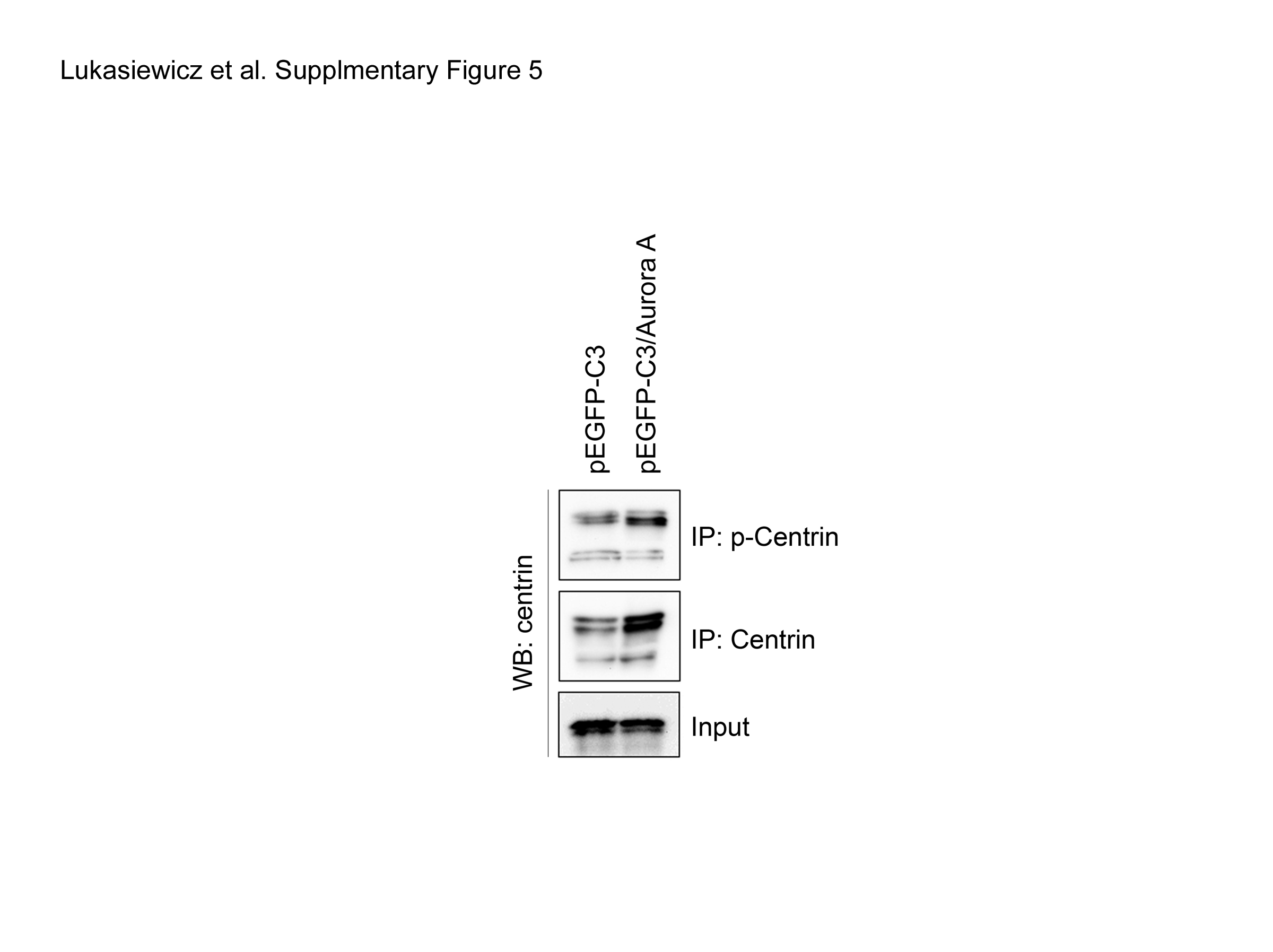

Supplement: Figure S5 — HeLa cells were transfected with either empty pEGFP-C3 or pEGFP-C3-Aurora A constructs and cells were harvested 48 hrs after transfection for immunoprecipitations using anti-centrin and anti-phospho-centrin antibodies. Immunoprecipitations were separated by SDS-PAGE and blotted with anti-centrin antibody. Note that there is more centrin and phospho-centrin in the pEGFP-C3-Aurora A expressing cells. (DOC) [file pone.0021291.s005.doc]
