# Supplementary material for: Control of Centrin Stability by Aurora A
Source: PLoS One. 2011 Jun 23;6(6):e21291. doi: 10.1371/journal.pone.0021291 (PMC3121746; doi:10.1371/journal.pone.0021291)

**Figure S6**: HeLa cells were processed for immunofluorescence as described in the Material and Methods section. Aurora A was detected with an anti-Aurora A antibody (green) and DNA was counterstained with DAPI (blue). An interphase cell is shown on the left side and an early prophase cell on the right side of the image. Note that Aurora A is much more abundant in the early prophase cell. Scale bar = 10 microns.


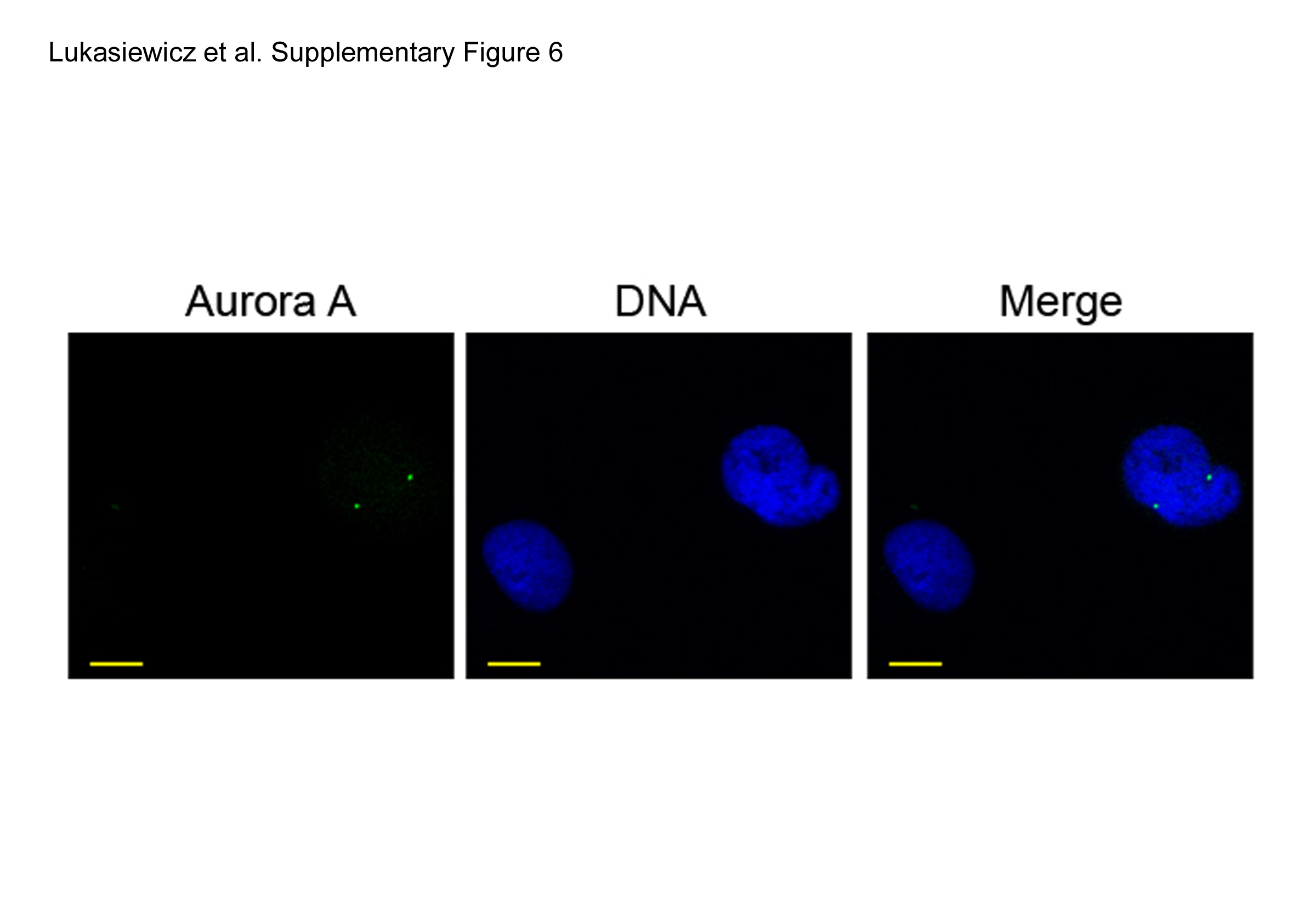

Supplement: Figure S6 — HeLa cells were processed for immunofluorescence as described in the Material and Methods section. Aurora A was detected with an anti-Aurora A antibody (green) and DNA was counterstained with DAPI (blue). An interphase cell is shown on the left side and an early prophase cell on the right side of the image. Note that Aurora A is much more abundant in the early prophase cell. Scale bar = 10 microns. (DOC) [file pone.0021291.s006.doc]
